# LysM Effectors: Secreted Proteins Supporting Fungal Life

**DOI:** 10.1371/journal.ppat.1003769

**Published:** 2013-12-12

**Authors:** Anja Kombrink, Bart P. H. J. Thomma

**Affiliations:** Laboratory of Phytopathology, Wageningen University, Wageningen, The Netherlands; Duke University Medical Center, United States of America

## Introduction

Fungi occupy a plethora of niches and play essential roles in diverse environments through decomposition of organic material as saprophytes or through establishment of symbiotic relationships with plants and animals that range from mutually beneficial to pathogenic. During colonization of their niches, fungi secrete proteins that include carbohydrate-degrading enzymes to feed on complex molecules and effectors that mediate the establishment of interactions with host organisms [1]. Although effectors are typically thought to be species- or even lineage-specific, some effectors are widespread among pathogens, such as the necrosis- and ethylene-inducing-like proteins (NLPs) that are widely spread in bacteria, fungi, and oomycetes [2], [3]. Several studies have shown that NLPs contribute to pathogen virulence through phytotoxic activity, but more recent work has revealed that some NLPs act in processes other than pathogenicity, such as fungal growth and sporulation [4]. A more recently identified class of conserved effectors are LysM effectors: fungal effectors that carry no recognizable protein domains other than lysin motifs (LysMs) [5]. Intriguingly, like NLPs, LysM effectors occur in both pathogenic and in nonpathogenic fungi.

## Plant Pathogen LysM Effectors: Virulence Factors through Interactions with Chitin

Microbial pathogens carry conserved structures, termed microbe-associated molecular patterns (MAMPs), that are recognized by host cell surface receptors and trigger an immune response [6], [7]. Chitin, a major constituent of fungal cell walls, is a well-described MAMP, and several plasma membrane–localized chitin receptors have been identified in plants that all contain extracellular LysMs, well-known carbohydrate-binding protein domains [8]–[10]. To overcome host immunity, genuine pathogens secrete effector molecules that manipulate host physiology, including immune responses, to support host colonization [2], [7]. Likely, also other microbes that establish intimate relationships with host plants, such as mutualistic symbiotic microbes and endophytes, secrete effectors to bring about their association.

**Figure 1:**
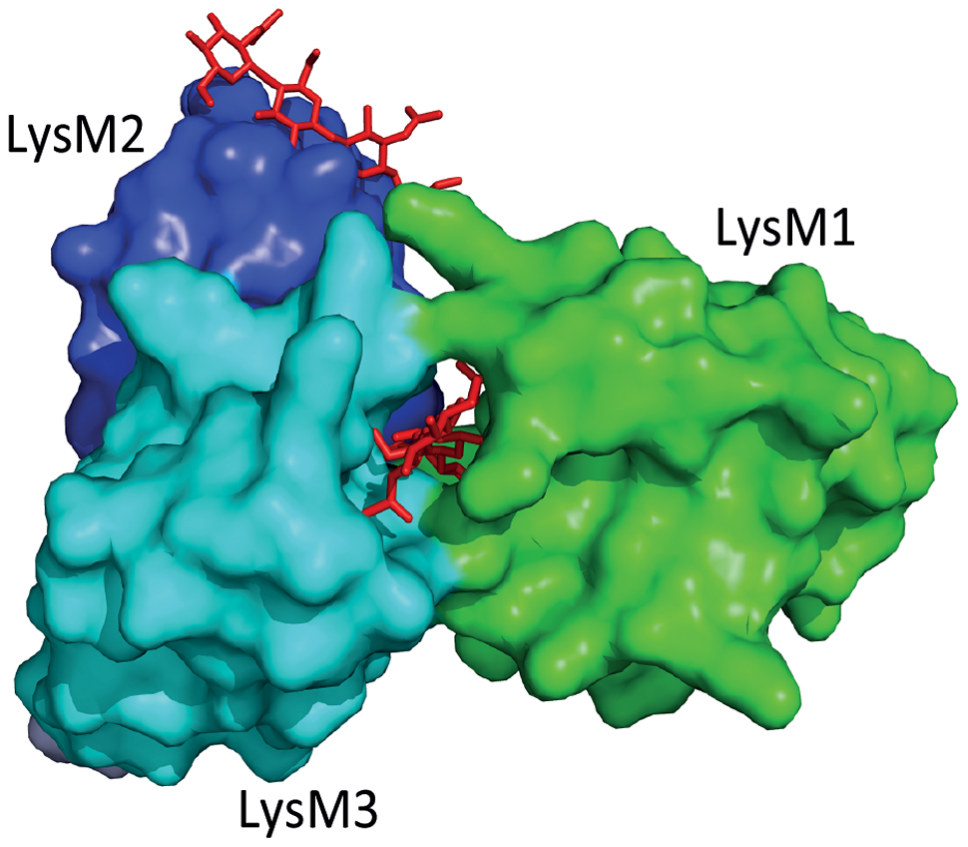
Three-dimensional structure of the *Cladosporium fulvum* LysM effector Ecp6. Two LysM domains of Ecp6 (LysM1 and LysM3) cooperate to form a binding groove that binds a single chitin oligosaccharide molecule (chitin tetramer oligosaccharide in red) with picomolar affinity. The remaining, singular LysM domain (LysM2) also has a functional chitin-binding site, although its affinity for chitin binding is significantly lower than that of the composite binding site.

**Figure 2:**
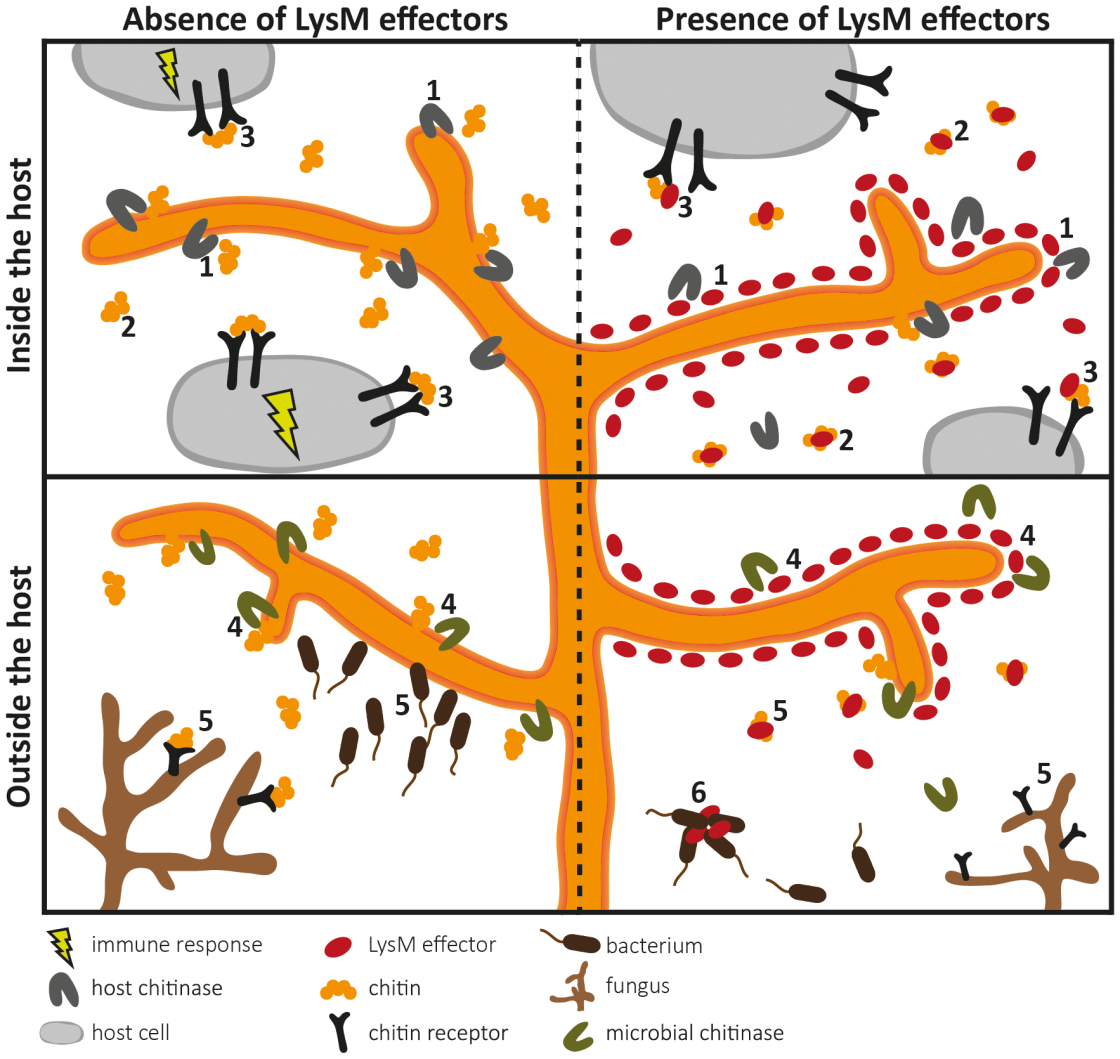
Overview of the diverse roles that fungal LysM effectors may play in fungal physiology. LysM effectors may act during host colonization (upper panels) and outside the host (lower panels). Pathogen LysM effectors have been implicated in two different pathogenicity-related processes (upper panels). Firstly, LysM effectors may protect fungal hyphae against degradation by hydrolytic enzymes secreted by the host (1). Secondly, LysM effectors may secure fungal cell wall–derived chitin fragments so that chitin cannot stimulate an immune response because LysM effectors efficiently scavenge chitin fragments (2), or interfere with host receptor activation by preventing ligand-induced dimerization (3). As LysM effectors also occur in nonpathogenic fungi (lower panels), they may protect fungal hyphae against hydrolytic enzymes secreted by mycoparasites (4). In addition, chitin sequestration might prevent attraction of such microbes (5). Some LysM effectors may recognize chitin-related carbohydrates such as peptidoglycan and immobilize bacterial competitors (6).

The fungal tomato leaf mould pathogen *Cladosporium fulvum* secretes the LysM-containing effector Ecp6 that binds chitin with high specificity [11], [12]. Ecp6 does not protect fungal hyphae against the hydrolytic activity of tomato chitinases, a function that was previously assigned to *C. fulvum* effector Avr4 that contains an invertebrate chitin-binding domain [13], [14]. Consequently, it was speculated that Ecp6 interferes with chitin detection by the host [5]. Indeed, Ecp6 was demonstrated to perturb chitin-induced immunity, and it was proposed that Ecp6 functions by sequestration of cell wall–derived chitin fragments that would otherwise be perceived by host immune receptors [12] (Figure 1). The crystal structure of Ecp6 showed that two LysM domains (LysM1 and LysM3) collectively bind a single chitin molecule [15] (Figure 1). This ligand-induced composite binding groove is deeply buried in the effector and displays ultra-high (picomolar) chitin-binding affinity, which is significantly higher than that of plant immune receptors [15]. Through analysis of a crystal structure of the *Arabidopsis* chitin elicitor receptor kinase (AtCERK1) it was previously demonstrated that only one of the three LysM domains in this immune receptor binds chitin [16]. Moreover, the structural orientation of the three LysM domains in AtCERK1 does not permit intramolecular LysM dimerization as observed in Ecp6 [15], [16]. Interestingly, the singular LysM domain of Ecp6 that is not involved in the intramolecular composite binding site (LysM2) also contains a functional chitin-binding site (Figure 1), and has the capacity to perturb chitin-induced immunity [12], [15]. Since the chitin-binding affinity of this singular LysM domain is significantly lower than that of the composite binding site, it is unlikely to deregulate chitin-induced immunity merely by chitin oligosaccharide sequestration. As it has been suggested that chitin-induced immune receptor dimerization is required for the activation of immune signalling, LysM2 may perturb chitin-induced immunity through interference with this dimerization [15], [16] (Figure 1, Figure 2). Since LysM effectors produced by the wheat blotch fungus *Mycosphaerella graminicola* and the rice blast pathogen *Magnaporthe oryzae*, Mg3LysM and Slp1 respectively, similarly suppress chitin-triggered immunity, it seems that deregulation of chitin-triggered immunity is an important function of LysM effectors [17], [18]. Nevertheless, functional analysis of *M. graminicola* LysM effectors has revealed that they may have additional functions during host colonization [10], [17]. Fungal cell wall chitin is a target of plant chitinases that act in fungal immunity; exochitinases release chitin oligosaccharide MAMPs from fungal cell walls that can induce host immune responses, which include the secretion of endochitinases that cause hyphal lysis [8], [19]. Interestingly, *M. graminicola* Mg1LysM and Mg3LysM prevent hyphal lysis by plant chitinases, whereas Ecp6 and Slp1 do not have this capacity [12], [17], [18] (Figure 2). Thus, functional diversification of LysM effectors during host colonization has occurred in plant pathogens.

## LysM Effectors as Virulence Factors of Mammalian Pathogens?

Genome mining revealed that LysM effectors are not confined to plant pathogens as, for instance, genomes of most (opportunistic) fungal pathogens of mammals contain LysM effector genes as well [5]. For instance, in the dermatophyte *Trichophyton rubrum*, causal agent of athlete's foot, as well as in related dermatophyte species, the gene family encoding LysM effectors appears to be expanded [20]. Similar to plants, mammals do not synthesize chitin but can respond to chitin with an immune response, which includes the production of chitinases [21]. These observations tempt speculation that fungal pathogens of mammals secrete LysM effectors to deal with host immunity in a similar fashion as plant pathogens [10]. Furthermore, allergies such as asthma are associated with fungal infections, although the underlying mechanisms presently remain unclear [22]. Emerging evidence suggests an important role for host chitinases that might mediate host responses to chitin and its derivatives [22], which may again be influenced by fungal LysM effectors. However, the fact that chitin is not universally recognized as a MAMP in mammalian systems argues against the hypothesis that fungal mammalian pathogens secrete LysM effectors to establish infection [23]. Furthermore, the genome of the human pathogenic yeast *Candida albicans*, as well as of most other *Candida* spp. that occur as opportunistic human pathogens, appears to lack LysM effector genes [5]. Similarly, in the genome of the skin-associated fungus *Malassezia globosa* that is responsible for the onset of dandruff and other skin disorders, and the fungus *Pneumocystis jirovecii* that causes pneumonia among immunocompromised hosts, no LysM effector genes are found [24], [25]. Since many mammalian pathogens show a low degree of host adaptation and lack host specificity, it has been suggested that infection by mammalian fungal pathogens does not require effector activity [1]. In contrast to plant pathogens, most fungal pathogens of mammals spend a considerable amount of their life cycle free-living in the environment and only infect mammalian hosts in an opportunistic manner. Thus, mammalian fungal pathogens may use their LysM effector homologues in processes other than host colonization, such as survival in the environment. The aforementioned absence of LysM effector genes in *Candida albicans*, *Malassezia globosa*, and *Pneumocystis jirovecii*, which are among the few fungal species that are commensals of humans and animals and that do not occur free-living in the environment, seems to support this hypothesis [5], [24], [25].

## LysM Effectors of Saprophytes: Diverse Possibilities

Considering that LysM effectors are ubiquitous in fungi, it could be argued that they might act in general physiological processes, such as cell wall modification. Fungi secrete lytic enzymes that break chitin polymers and in this manner maintain cell wall flexibility to allow hyphal growth, branching, morphogenesis, and spore germination. Recently, a *Trichoderma atroviride* LysM effector was found to be coexpressed with an adjacent chitinase gene [26]. Since addition of the purified LysM effector to *T. atroviride* inhibited spore germination *in vitro*, a role in hyphal growth was proposed for this LysM effector. However, further experimental evidence that includes targeted deletion of the LysM effector gene in *T. atroviride* is required to support such a role. Their occurrence in saprophytes may furthermore suggest that LysM effectors contribute to growth in any fungal niche, as likely other microbes are encountered that compete for the same niche or may act as mycoparasites. In this respect, several hypotheses can be envisaged. Extrapolating the findings for LysM effectors of plant pathogens, LysM effectors may protect fungi against chitinases and other hydrolytic enzymes produced by mycoparasites. Moreover, sequestration of cell wall–derived chitin oligosaccharides may be relevant if mycoparasites would be attracted by gradients of such fragments (Figure 2). One step further, LysM effectors may also have functions that are not associated with chitin binding. Originally, LysMs were identified in bacterial lysozymes (hence the name of the domain) that bind and hydrolyse peptidoglycan, a chitin-related glycan and a major component of bacterial cell walls [27]. LysMs occur in various peptidoglycan-binding proteins, and thus it is conceivable that some LysM effectors bind peptidoglycan as well. Such LysM effectors may help fungi to affect bacterial competitors in their niches, for instance because they immobilize them in a similar fashion as antibodies do [28] (Figure 2).

## Concluding Remarks

Fungal LysM effectors are versatile proteins that occur in fungal species with extremely divergent lifestyles. Conceivably, LysM effectors function in various ecological niches. In addition, even LysM effectors of plant pathogens that function in the same niche (the plant host) and that bind the same substrate (chitin) were demonstrated to have distinct roles in promoting fungal virulence [10], [17]. Furthermore, pathogens interact with other microbes, both in the free-living stage and during colonization of their hosts where they may encounter opportunistic pathogens, commensals, and endophytes. In this respect it is interesting to note that strains of the vascular wilt pathogen *Verticillium dahliae* have a significantly expanded LysM effector family of six to seven members [29], [30]. However, functional analysis has revealed that only one of these LysM effectors is induced *in planta* and contributes to pathogenicity, while the role of the others still remains obscure [30]. *V. dahliae* is known to survive as a resting structure in the soil for decades in the absence of suitable host plants, and it is tempting to speculate that its LysM effectors contribute to persistence of these structures through protection against microbial activity. Therefore, the study of LysM effectors of fungi that thrive in a variety of niches will reveal additional LysM effector functions that are relevant for pathogenic fungi as well.
